# Supplementary material for: Content-rich biological network constructed by mining PubMed abstracts
Source: BMC Bioinformatics. 2004 Oct 8;5:147. doi: 10.1186/1471-2105-5-147 (PMC528731; doi:10.1186/1471-2105-5-147)
Supplement: Additional File 2 — The original results of the above study (non-essential files are deleted to keep the file size under the limit set by BMC bioinformatics). [file 1471-2105-5-147-S2.bz2 › chilibotAdditionalFile2/dip05/703ID10809785E18036/html/MALF_MALK.html]

 


 **MALF** and **MALK** 
  
Found 40 abstracts in PubMed, retrieved 05.  
 

 What does Google say? 
 PDF only 
| .edu only 

---

**Interactive relationship** (e.g. stimulation, inhibition, etc)

**Neutral relationship**- The maltose transport complex of Escherichia coli, a member of the ATP binding cassette ABC superfamily, is made up of two nucleotide binding subunits,  **MalK**  2, which hydrolyze ATP with positive cooperativity, and two transmembrane subunits,  **MalF**  and MalG.  Ref: 12093921 Proc Natl Acad Sci U S A, 2002

**Non-interactive relationship** (e.g. studied together, co-existance, homology, etc.)

- We report the characterization of TrmB, a protein of 3 00 apparent molecular weight, that is involved in the maltose specific regulation of a gene cluster in Thermococcus litoralis, malE  **malF**  malG orf trmB  **malK** , encoding a binding protein dependent ABC transporter for trehalose and maltose.  Ref: 12426307 J Biol Chem, 2003
- First, we optimized the yield in purified complex protein by taking advantage of a newly constructed expression plasmid that carries the  **malK** ,  **malF**  and malG genes in tandem orientation.  Ref: 12225853 Biochim Biophys Acta, 2002
